# Superstitious conditioning shapes the illusory experience of free will under causal determinism

**DOI:** 10.3389/fnhum.2026.1832135

**Published:** 2026-05-26

**Authors:** Cooper Kansala, Emre Cicek, Vanessa B. Nkansah-Okoree, Andrew W. Golding, Nirosha J. Murugan, Nicolas Rouleau

**Affiliations:** 1 Department of Health Sciences, Wilfrid Laurier University, Waterloo, ON, Canada; 2 The Allen Discovery Center, Tufts University, Medford, MA, United States; 3 Department of Biomedical Engineering, Tufts University, Medford, MA, United States

**Keywords:** free will, dopamine, causality, misattribution, premotor cortex, superstitious conditioning

## Abstract

Discussions of free will often center on the question of whether actions are determined, with a special focus on causes, effects, and *when* subjects experience the desire to act relative to neural events. However, because the only evidence for the existence of free will is the subjective experience itself, explanation must center on the phenomenal: why do we *feel* free? Here, we propose a neuropsychological model of free will as a conditioned illusion—a misattribution of causality reinforced through operant learning. First, we postulate that decisions and actions are determined. Then, drawing on classical findings such as the readiness potential preceding conscious intention and more recent work on active inference, we propose that the temporal contiguity of premotor and motor activations gives rise to superstitious conditioning, shaping an experience of—and belief in—free will that is compatible with determinism. Sustained by dopaminergic circuits and reinforced by sensorimotor feedback loops, our framework situates free will as an experiential phenomenon rooted in the functional neuroanatomy of learning. We discuss implications for medicine, ethics, law, and suggest conditions under which the experience of free will may be disrupted or restructured by disease, pharmacology, and reinforcement history.

## Introduction

Central to human experience is the intuition that our actions are consciously and deliberately chosen from a set of possible options—that we are free to say and do as we will in any given moment. The experience of free will is often associated with the belief that if one could rewind time and revisit past decisions, all else being equal, one could choose a different course of action, free of any former constraints or influences. While some individuals believe their actions are determined by luck, fate, or other people and events outside of their control (Specht et al., 2011), most report the experience of a potent, internal locus of control. That is, most people feel they are the conscious authors of their own actions—a belief that positively correlates with gray matter volume within the anterior cingulate cortex, striatum, and anterior insula (Hashimoto et al., 2015). Belief in free will also correlates with certain behavioural outcomes (Baumeister et al., 2009). When individuals were primed to believe their actions were determined, they displayed more cheating behaviours relative to those who were primed to believe their actions were free from external influences (Vohs and Schooler, 2008). Manipulations such as these support the position that neural events and their cognitive-behavioural correlates are determined by prior causes, the vast majority of which remain inaccessible to conscious awareness. Neuroimaging studies have repeatedly demonstrated that decisions and actions can be predicted several seconds before conscious awareness of the decision itself (Libet et al., 1993; Soon et al., 2008). The experience of free will exists, as it were, in the wake of unsupervised processes that are predictive of future actions. However, there is yet no mechanism to explain how such an experience might arise in the absence of free will.

The assumption that effects in the future can be predicted by knowledge of prior causes is fundamental to the metaphysical view of causal determinism. The French mathematician and astronomer Pierre-Simon de Laplace best illustrated this principle by imagining a creature now known as Laplace’s demon (Kožnjak, 2015) that, given complete knowledge of the position and motion of all particles in any moment, could know the future with certainty (Van Strien, 2014). In practice, however, even a determined system can escape prediction (Shermer, 1995; Van Kampen, 1991). And while some have argued that discoveries in quantum physics challenge deterministic models of the Universe (Falkenburg and Weinert, 2009; Esfeld, 2000) and the mind (Hameroff and Penrose, 1998; Hameroff, 2012), it remains true that from the perspective of an external observer, human thought and behaviour can be both predicted and controlled under the assumptions of causal determinism (Bargh and Ferguson, 2000). Indeed, the behaviourist school, with its highly reliable methods of predicting and controlling animal behaviour, was fundamentally grounded in a broader deterministic framework. Within this context, two popular philosophical positions have emerged as plausible accounts of the veridical status or objective existence of free will: determinism (Van Inwagen, 1975) and compatibilism (Van Inwagen, 2008). A comprehensive review of these contrasting positions is beyond the scope of the current paper because our goal is not to build a case for or against the existence of free will as such, nor is it to recapitulate an even-handed debate on the topic. Rather, our goal is to describe how the experience of free will is formed and maintained if, in fact, decisions and actions are bound by causal determinism. We are focused on this question because, if the primary evidence for the existence of free will is the subjective experience of it, a description of how such an experience can exist in a deterministic world is tantamount to an explanation of the full phenomenon. While we acknowledge the ongoing debate about the existence of free will, here, we postulate rather than conclude that decisions and actions are determined and then hypothesize a mechanism for the persistence of a psychologically normal but fundamentally illusory experience of free will.

## The incoherence of free will under causal determinism

While we are treating causal determinism as a postulate rather than a conclusion, a brief review of the relevant terms and concepts is in order. The determinist position holds that cognition and behaviour are caused by a continuous chain of prior events whose ultimate origin is not conscious and unlikely to localize strictly within the body. Determinists argue that the neural events that are the proximate causes of decisions or other actions are themselves not chosen or accounted for by the subject. In a deterministic world, environmental (e.g., sounds, sights, smells, tastes) and internal (e.g., memories, desires, conditioning) influences arise spontaneously (though, predictively) to orchestrate brain functions, including those that govern decisions and actions, of which we later become aware. This view is consistent with the observation that neural events predictive of future actions arise *upstream* of the conscious experience of free will (Libet et al., 1993; Soon et al., 2008). A determinist views the experience of free will as an illusion (Harris, 2012; Sapolsky, 2023)—a passive state of observation mistaken for intentional action with real causal power.

Some have argued that if subjects are their whole bodies, including their predominantly unconscious nervous systems, willed actions can be attributed to subjects themselves (Dennett, 2003). Others have rejected the view that free will is not a physics-defying independence from causality (indeterminism) and agency can exist despite causal determinism (Mitchell, 2023). This “compatibilist” view—of which there are several subtypes—is said to reconcile free will within the bounds of causal determinism by extending concepts of self and agency to include every part of the human organism, including those which are most subjectively inaccessible (Dennett, 2003). Crucially, the compatibilist view resolves problems of moral responsibility that can be difficult to accommodate under a deterministic framework (Fischer and Ravizza, 1998). Of course, most people do not identify with their spinal cord or hypothalamus, not to mention their gut microflora or appendix—all of which nevertheless contribute directly or indirectly to the organism’s thoughts and actions. Indeed, the conscious, waking experience of a self that “decides” and “acts” is, in large part, a cortical phenomenon (Krawczyk, 2002) with necessary contributions from subcortical (Redinbaugh et al., 2020) and brainstem nuclei (Parvizi and Damasio, 2001). More importantly, the potency of what most people mean by free will is greatly diminished if decisions are made by unconscious brain processes before the subjective experiences of decisions and actions arise. Whether it’s an accurate description or not, the free will of compatibilism is somewhat out of touch with the felt experience of being an agent somewhere behind the eyes and midway between the ears, consciously authoring actions in real-time.

While determinists and compatibilists disagree about the veridical status of free will, both accept the existence of a conscious *experience* of free will. This distinction is important, because unlike truth claims about the existence of an ability to act from internal causes independent of external influences (i.e., uncaused cause), the existence of an experience of free will cannot be doubted on the basis of causal determinism. Whether they are free or not, humans report a felt freedom to walk, talk, gesture, and gaze in ways that are consistent with their privately experienced desires, goals, and values. There is nothing about determinism that makes these self-reported experiences unexpected. Other actions, such as arm-swing, microexpressions, compulsions, tics, and dozens of reflexes are experienced as involuntary—which is to say, some actions feel unplanned by the subject, though motivations to act are often inferred or rationalized after the fact (Cushman, 2020). Breathing is one of a handful of actions that, from the perspective of the subject, straddles a line between the voluntary and involuntary (Hudson et al., 2011). Indeed, the subject’s attention to their own breathing seems to suddenly place the otherwise automatic motion of the diaphragm under “conscious control” (Brown and Gerbarg, 2009). When attention wanes, automaticity seems to resume. It has been suggested that this particular experience, which is central to some meditation practices, exposes the experience of free will as a misattribution of effect for cause (Watts, 2010). Demonstrating this phenomenon experimentally, respiration recordings were mapped on to a digital avatar in an immersive virtual reality space (Monti et al., 2020). Observers experienced an illusory sense that the virtual body was their own and that they were in control of the avatar. This effect, termed “embreathment,” was dependent upon sensory feedback from a virtual body that was more human-like. In other words, directing attention to breathing and receiving feedback evokes an illusory experience of conscious control.

Interestingly, it was demonstrated that human participants who were unwittingly exposed to transcranial magnetic stimulation (TMS) of their motor cortices, when asked to move either their left or right finger, could be reliably induced to move the finger contralateral to the stimulated hemisphere without affecting their experience of having made a choice free from external influences (Brasil-Neto et al., 1992). In other words, the authors found that they could control the actions of their subjects with neurostimulation without impacting the experience of free will and concluded that the experienced decision to move is likely intrinsic to the process of movement itself. That, from the perspective of the subject, the will to move seems to precede the movement is irrelevant to whether or not the events share an objective cause. Consider the analogy of observing lightning: The flash of light that appears to precede thunder is not the cause of thunder. Both photon emissions and mechanical vibrations of air are caused by a third factor: a sudden discharge of electric current. Here, the flash and the bang are parallel effects of a shared prior cause. However, because light travels nearly 1 million times faster than sound, and the observer perceives the flash before the bang, it is trivially easy for an observer to commit a misattribution error. In principle, this type of error is possible for any two events presented in rapid succession where causality is assumed on the basis of temporal proximity. Therefore, the conscious experience of “choice” preceding action does not necessarily imply any cause-and-effect relationship. Rather, the evidence suggests the opposite temporal sequence (Libet et al., 1993; Soon et al., 2008) or perhaps that these events co-occur (Brasil-Neto et al., 1992). Under causal determinism, where prior causes predict future events, free will is an incoherent concept; however, because the arrow of time cannot (yet) be reversed and total entropy is always increasing, the hypothesis may be untestable in principle, as others have suggested (Northcott, 2019). Still, the existence of a persistent experience of free will under the constraints of causal determinism demands an explanation.

## Why do we feel free? A hypothesis

If our decisions and actions are indeterminate, our experience of free will is in alignment with reality; however, if they are determined, the very same experience is an illusion. Why do we *feel* free? The question of why we experience free will is, of course, more tractable than the issue of whether we are free to make conscious choices. However, a passive experience of free will, stripped of causal impact, does not seem to add value to the decision-making process. Is there any added predictive value to a model of animal decision-making that inserts a conscious but impotent subject at some point along the causal chain between prior causes and present actions? Of course, sensory-motor feedback loops can and do modify behaviour (Codol et al., 2023; Pezzulo et al., 2015). But does the mere added presence of a subjective experience, whose states are also determined by prior causes, change the expected outcomes of the system in any way? How would an internal observer of pre-determined events have any more impact on those passing decisions and actions than an external observer?

From the perspective of the external observer, decisions and actions are sufficiently explained by the activities of embodied brains (Kiverstein and Miller, 2015; Linson et al., 2018; Robertson and Kirchoff, 2019; Kagan et al., 2022), independent of their subjective states. Early behaviourists demonstrated that the assumption of a mind was not necessary to measure, predict, and control behaviour (Moore, 1981; Mills and Mos, 1999). Indeed, contemporary evidence also suggests that subjective experience is not needed to display intelligent behaviour (Dreyfus, 2002). For example, neuronal monolayers on microelectrode arrays display problem-solving when placed in a closed-loop feedback system (Kagan et al., 2022; Rouleau and Levin, 2023; Rouleau et al., 2021). While cultured monolayers may be sentient, there is yet no clear evidence of subjective experience in a dish (Rouleau and Levin, 2023; Rouleau et al., 2021). However, so-called agential systems are often described as if they are conscious “movers”; which is to say, subjects that act by virtue of their experiences as causes rather than effects or coincidences. Is there ever any predictive value of adding subjects to physical models of, say, the motions of tectonic plates or weather patterns? Why do we feel compelled to add a conscious mover into models of human psychology when awareness of decisions arises downstream of the determinants of decision-making and behaviour can be explained and controlled without the assumption of subjectivity? It is said that when Laplace was asked by Napoleon about the omission of God from his model of the solar system and celestial mechanics, he simply stated that the hypothesis was not required (Hankins, 2006). While likely apocryphal, this well-known anecdote highlights the general principle that useful models need only include factors that maximize predictive power, and nothing more.

Consciousness, which we understand to be synonymous with a capacity for subjective experience, is not an effector—it does not directly act upon the world. There should be little doubt that consciousness exists as a perceptual phenomenon—experience is our only point of access to the external world. What we are calling into question is its causal potency, reflected as directed actions in a world external to itself. Because the conscious experience of free will arises after neural activity predictive of future actions, it is unlikely to hold causal power in the sensorimotor loop. In the case of free will, we argue that actions in the objective world and the subjective experience of free will are both explained without the incoherent hypothesis of free will. Here, we present an alternative hypothesis—that the experience of free will is (1) coincident with the determinants of actions, (2) a misattribution error, and (3) is formed, reinforced, and sustained by superstitious conditioning. We claim that once the illusion of free will is experienced, a belief in free will is formed that is projected to erroneously infer its existence in others. In addition to a description of its mechanism, we will discuss the neuropsychological correlates of the experience of free will and its absence under certain conditions. Finally, we will briefly explore the implications of our hypothesis in medicine, ethics, law, and beyond.

## Superstitious conditioning shapes belief and behaviour

A superstition is a belief that is formed from a false conception of causation (Griffiths et al., 2019). Superstitions involve avoiding or engaging in actions that are expected to produce events, which always occur after the action and are perceived as causal outcomes. These beliefs, which are often culturally dependent, persist within individuals and groups despite a lack of evidence or even a plausible causal link between the event and the outcome. In Western cultures, some actions are believed to potentiate negative events, such as spilling salt or breaking mirrors, while positive events are associated with the discovery or use of objects such as four-leaf clovers and horseshoes. However, superstitions are, first and foremost, beliefs (Maller and Lundeen, 1933)—they are privately held convictions that can but need not necessarily manifest as behaviour in any given moment. Many private beliefs, held by humans everywhere on Earth, are fundamentally superstitious, including concepts of luck, fate, destiny, omens, and karma (Exline and Wilt, 2023). It should be noted that superstitious thinking is not pathological and that most humans hold one or more irrational beliefs of this kind. Could the experience of free will give rise to yet another type of superstitious belief?

In his 1948 paper entitled “Superstition in the pigeon”, B. F. Skinner remarked that conditioning occurs because of the temporal sequence of response and reinforcement (Skinner, 1948). As he argued, all that is required for an organism to be conditioned is for reinforcement to follow a response. The presence of an experimenter or external observer is not necessary. Indeed, any machine, organism, or natural event may act as a mediator for the delivery of the reinforcement that shapes the learned response, so long as the temporal sequence is preserved. Skinner cites several experiments with pigeons as evidence for learned superstition, with a particular emphasis on the importance of fixed intervals of time between the action and the event, with much greater effectiveness for shorter intervals (Skinner, 1948). In the classic example of “non-contingent reinforcement” (Holden, 2005; Aeschleman et al., 2003), to enhance the reinforcing properties of food delivery, a pigeon’s access to food is first restricted until its weight is reduced to 75% of its counterparts’ that are fed *ad libitum* (Skinner, 1948). Then, the animal is given access to a custom hopper apparatus that it can peck to produce food after a 5-s delay. Because pecking is reliably paired with food delivery after some delay, the pigeon will continue to engage in pecking-for-food behaviour. However, if the hopper is instead controlled by a clock that delivers food at regular intervals to the pigeon without reference to its behaviour (i.e., non-contingency), conditioning occurs all the same. Remarkably, the reinforced behaviour is whichever action happened to be displayed immediately before the food was delivered, which can vary greatly. Indeed, pigeons displayed unique superstitious responses such as lifting invisible bars, thrusting their heads into the corners of their cages, rotating their bodies, pecking the air, or swinging their heads (Skinner, 1948). All that was required for the superstition to be formed was for the previously random action to be displayed immediately before the delivery of the reinforcement. Once reinforced, the behaviour was displayed repeatedly, including before subsequent reinforcements at fixed intervals, thus sustaining the behaviour.

Of course, superstitions can be passed down generationally or transmitted laterally through the culture without the superstitious individual being directly conditioned. Consider the naturalistic examples of rainmaking (Moore and Burgess, 2011; Seneviratna, 1984) and animal sacrifice (De Farias, 2020), which are sometimes observed together (Larson, 1966). Faced with famine, drought, and other types of natural disasters, humans in search of solutions to existential threats have historically displayed increasingly desperate behaviours, informed by cultural expectations. Negotiations with a hostile environment may start with subtle behaviours such as prayer or wishful thinking; however, with time, more extreme actions will be taken, often as acts of devotion. Sacrifices and other rituals, which are displayed later in the timeline of the natural disaster as conventional approaches fail to produce expected results, are more likely to be displayed immediately before relief. In other words, because it will eventually rain, extreme behaviours displayed later in the drought can be non-contingently reinforced (i.e., raining occurs independent of but in sequence with individual human behaviours) to form new superstitious beliefs such as dances, chants, sacrifices, and other “rainmaking” behaviours that are perceived as causes of rainfall because they immediately preceded relief. It should be noted that removing an unpleasant or aversive stimulus (i.e., relief by withdrawal of negative reinforcement), like the addition of a positive reinforcement, will promote more frequent displays of the preceding behaviour. We will argue that the experience of free will can be similarly shaped by neural circuits governing superstitious conditioning and the reinforcement of predicted future actions.

The neural correlates of superstitious conditioning and beliefs center on two major functional subdivisions of the brain. The first and most conspicuous correlate is the mesolimbic or “reward” pathway and other closely related dopaminergic pathways, which include structures of the midbrain tegmentum, basal forebrain, ventral striatum, and prefrontal cortex (Pierce and Kumaresan, 2006; Nestler and Carlezon Jr, 2006). Indeed, it has long been hypothesized that delusions and superstitious thinking are related to dopamine signaling (Shaner, 1999). We now know that superstitions are less commonly held by patients with Parkinson’s Disease (PD)—a disorder characterized by marked reduction of the dopamine-cell-producing substantia nigra—than those with epilepsy, multiple sclerosis, or healthy controls (Mameniškienė et al., 2024). On the other hand, individuals with schizophrenia—a condition defined in part by delusions related to causality as well as “forced” thought and actions (e.g., “X, not me, made me do it”) that can be inhibited by administering dopamine blockers—are far more likely than their healthy counterparts to display superstitious beliefs (Shiah et al., 2014). It may be relevant that chronic administration of ketamine, a psychomimetic drug, increased the propensity of healthy volunteers to adopt superstitious thinking (Freeman et al., 2009). Notably, dopamine pathways are integral to motor function—particularly those described as “spontaneous” and “involuntary,” such as arm swing, laughter, and gait (Selikhova et al., 2008). Of course, most people can swing their arms, laugh, and walk with the experience of a potent free will; however, the perceived automaticity of these extrapyramidal events is worth noting given the role of the basal ganglia and dopaminergic pathways in superstitious conditioning. Incidentally, the same dopamine pathways associated with superstitious conditioning are integral to “decision-making” in terms of embodied or active inference (Friston et al., 2014) as well as forming and sustaining addiction, which is often experienced as a disturbance of will.

The mesocortical dopamine pathway serves as a major input from the dopamine-rich ventral tegmental area to the prefrontal cortex, which is intimately connected to the second major correlate: the premotor cortex. Rao et al. conducted an MRI study to evaluate superstitious decision-making tied to beliefs in auspicious dates, identifying the right middle and superior frontal gyri as major determinants of participants’ actions (Rao et al., 2014). It should be noted that the posterior portions of both of these gyri constitute the premotor cortices—the areas conventionally described as “organizing” and “planning” centers that determine downstream actions executed by the motor strip. Grössinger et al. used a sham neurofeedback task to induce patients to believe that they were in control of moving bars on a screen that were not coupled to any brain activity (Grössinger et al., 2021). The authors reported strong activation of the basal ganglia, ventral striatum, anterior insula, supplementary motor area, and middle frontal gyrus—a canonical premotor region—associated with the superstitious control. That is to say, when participants experienced free will of movement despite lacking any true control (superstitious misattribution of causality), their premotor cortex—conventionally understood to plan willed movement—was among those regions which were most activated. These data indicate that superstitious conditioning is intimately tied to dopaminergic pathways as well as activity within the premotor cortices and basal ganglia that have been conventionally linked to “voluntary” decisions and actions.

### A neural mechanism for the free will experience

The neuropsychological correlates of free will were first delineated by Benjamin Libet (Libet et al., 1993), whose seminal experiments demonstrated that the conscious urge to initiate movement was reliably preceded by activity—or a ‘readiness potential’—within areas of the frontal lobes anterior to the motor strip. These areas, often referred to as “premotor” or “supplementary” to motor cortices, correspond to the posterior-most regions of the superior, middle, and inferior frontal gyri that border the precentral sulcus (Crosby et al., 1955). From a functionalist perspective, they are the proximate causes of upper motor neuron activity. Libet found that hundreds of milliseconds before participants were conscious of their will to initiate movement, the functional states of their brains were already announcing the inevitability of a particular action (Libet et al., 1993). Of course, Libet’s experiments have been criticized as simplistic, arbitrary, or contrived (Schurger et al., 2025; Maoz, 2023), and there is reason to believe that the readiness potential does not represent anything like a true initiation point with alternative interpretations of the decision-making timeline (Schlegel et al., 2011). However, we later learned that decision outcomes are encoded by neural activity within the prefrontal and parietal cortices at least 7–10 s before any conscious experience of willed action (Soon et al., 2008). This revised threshold may be reflective of the current limits of our neuroimaging technologies and statistical tools rather than some definitive time point where decisions are *truly* initiated. Consistent with these findings, some authors have suggested that the greater prefrontal cortex should be considered “premotor,” representing a significant functional expansion of the definition (Fine and Hayden, 2022). Under causal determinism, however, any neuronal activity displayed 10 or any number of seconds before the conscious experience of free will is hostage to a chain of prior causes that predict future events. Therefore, we anticipate that with the application of future machine learning techniques to identify the subtle signal features consistently displayed before decisions to act, predictions of decision outcomes will be possible at even earlier timepoints—perhaps minutes before the decision if the experimental design is appropriate. Further, we expect that the brain regions within which activity predicts decision outcomes will become more diffuse, incorporating neural substrates with increasingly indirect connections to the motor strip and its immediate proximate afferents in the premotor areas. What is the experiential equivalent of this extended premotor response?

**Figure 1:**
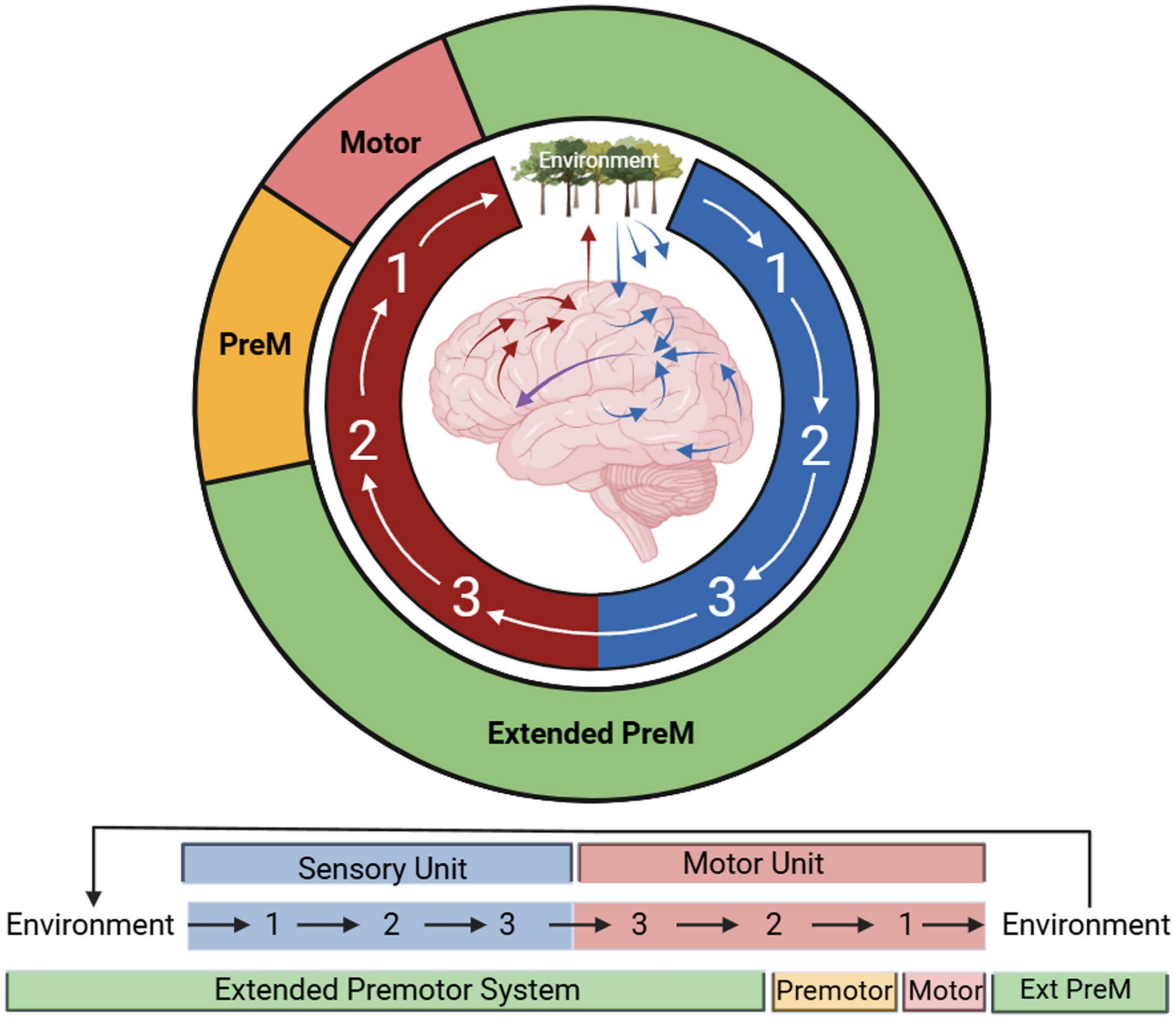
Model of the extended premotor system within the sensorimotor loop under causal determinism. The model illustrates how sensory information from the environment (blue pathway) is processed by the sensory unit (1, primary cortex, 2, secondary association cortex, 3, tertiary association cortex) before interfacing with the motor unit (red pathway) to generate actions (3, 2, and 1 refer to the reverse sequence of cortical activations preceding actions), which in turn influence the environment and sensory feedback. Sensory and motor units are embedded within an extended premotor (Extended PreM) system (green) that determines future neural events far beyond the conventional premotor (PreM) regions anterior to the precentral sulcus. Although behaviour is driven by a causal chain of events extending beyond the individual (i.e., the environment-organism system), subjective experience of free will is proposed to emerge specifically at the premotor-motor interface, where predicted actions are realized, manifest in the external world as bodily actions and interactions with objects. Arrows superimposed over the brain reflect a hierarchical organization of sensory processing (blue), conduction to the motor unit (purple), and proximate determinants of motor activity (red); however, we propose that the full sensorimotor loop is “premotor,” determining outcomes well before any conscious experience of free will or action. This figure was created in BioRender.

The events immediately preceding an action experienced as voluntary are often narrativized as beginning with “decisions,” followed by “planning,” and finally, the execution of motor sequences. Here, there is an implicit assumption of free will, where the decision to act is spontaneously generated without prior cause. Typically, the implicated functional neuroanatomy includes large swaths of the frontal lobes and anterior cingulate gyri with increasingly caudal activations across frontal (i.e., premotor) regions toward the precentral gyrus (i.e., the motor strip, BA4), where upper motor neurons directly determine bodily actions (Rizzolatti and Luppino, 2001; Peña-Casanova et al., 2024). What are the prior causes of premotor activity? Of course, the frontal gyri receive direct afferents from the parietal, cingulate, temporal, insular, and occipital cortices, as well as thalamic nuclei and the basal ganglia (Crosby et al., 1955). However, in terms of degrees of separation, neurons governing premotor function are only a handful of synapses away from every other nucleus or cortical region in the central nervous system. Therefore, within the chain of causes preceding actions, the prefrontal cortex is only the most proximate premotor region. As illustrated by hierarchical models of cortical function (Peña-Casanova et al., 2024; Pąhalska and Kaczmarek, 2012), where information flows from sensory to motor units, the cortex is effectively a “premotor” organ. Consistent with the function of sensorimotor feedback loops and the principles of active inference (Pezzulo et al., 2015; Kiverstein and Miller, 2015), the environment must also be factored into the causal chain. Indeed, in our view which is compatible with causal determinism, premotor events include environmental stimuli, sensory organ activation, brain stem and thalamic relays, processing in primary receptive and secondary associative cortices, tertiary encoding associated with language and the sense of self, prefrontal activations, and classical premotor readiness potentials (Figure 1). As these premotor events ultimately impinge upon upper motor neurons, whose precise outputs are determined by the targets of efferent fibers, the embodied brain interacts with and changes its environment, thus renewing the sensorimotor feedback loop.

If brain-environment interactions are mediated by sensorimotor feedback loops with an extensive, intervening premotor phase that predicts future actions, all that would be required to generate the experience free will would be to misattribute the anticipation of inevitable motor events for conscious control. Because premotor events, which cause future actions, precede both the decision to act and the action itself, there must be some mechanism which convinces the subject that the premotor events were selected rather than simply arising as part of the extended sensorimotor loop. Here, superstitious conditioning bridges the explanatory gap. If part of the premotor phase involves prediction of future events, perhaps the realization of that prediction by a comparator is intrinsically reinforcing. It has been suggested that the reduction of surprise or uncertainty is a major driver of living and self-organizing systems, including the brain (Parr and Friston, 2017). Indeed, neurons and their networked aggregates build internal models (Pezzulo et al., 2024), self-reinforced by looped feedback, to maximize prediction accuracy (or minimize prediction error). Because premotor events always precede motor events, and the subject is only ever aware of the motor event itself, perhaps the temporal sequence of premotor-motor pairings is consciously experienced as free will. Under these conditions, the subject feels free—they know exactly what they’ll do next, not because they willed it, but because they have access to the premotor information content (via internal models) that predicts downstream events (unconscious inferences), which is always reinforced by the conscious experience of embodied actions that follow motor strip activation. In other words, the experience of free will is a misattribution error committed by a subject whose experience, initially defined by the premotor state of the brain, suddenly becomes witness to the realization of predicted actions which were always inevitable. The self-perceived “agent” arises spontaneously within the sensorimotor loop, at the interface of premotor and motor events, conditioned to experience actions and their environmental effects (e.g., bodily movement, forces applied to objects, etc.) as willed rather than witnessed. Because superstitious conditioning is a type of learning, perhaps there are certain conditions by which premotor predictions and experienced actions can be uncoupled, thus “breaking the spell” of experienced free will.

## Breaking the spell

Under what conditions do humans report the absence of an experience of free will? As we touched on previously, individuals with psychiatric and neurological disorders characterized by dysfunctions of the basal ganglia, dopaminergic pathways, frontostriatal circuits, and premotor cortices report a range of experiences marked by involuntary or exogenous thoughts and actions. These include individuals with movement disorders, schizophrenia, addiction, attention deficit hyperactivity disorder (ADHD), and obsessive-compulsive disorder (OCD) (Pierre, 2014; Vonasch et al., 2017; Meynen, 2010). It is a well-known clinical phenomenon that PD patients treated with dopamine receptor agonists to regain “voluntary” control over their bodies can develop pathological gambling habits and other addictions that compel the individual to act impulsively (i.e., disorders of will) (Stamey and Jankovic, 2008; Voon et al., 2011). Children and adults diagnosed with ADHD—a disorder treated with stimulants that inhibit the reuptake of catecholamines including dopamine—are disproportionately likely to report an external locus of control (Lufi and Parish-Plass, 1995; Rucklidge et al., 2007; Rucklidge and Kaplan, 1997). Among patients diagnosed with schizophrenia, the experience of free will was inversely related to the severity of their positive symptoms, which include delusions and hallucinations (Weisman de Mamani et al., 2016)—the very same symptoms that are inhibited by the administration of dopamine receptor antagonists. Consistent with involvement of the mesolimbic dopamine system, there is also evidence to suggest that features of both avolition and abulia—a state of diminished motivation to act—can be learned.

Acquired avolition has been modeled in humans and rodents using reward-based behavioural paradigms, revealing a profound impact on the “wanting” experience, which is a deficit of anticipatory pleasure (DeRosse et al., 2019). Being unable to predict incoming reinforcements diminishes desire, causing a lack of persistent goal-directed behaviour. This could suggest an intrinsic motivating force of circuits driving active inference processes (Millidge et al., 2021). Similarly, animals will display learned helplessness when exposed to non-contingent negative reinforcement (Hiroto, 1974). Classically, if an animal cannot escape or avoid an aversive stimulus (e.g., shocked, scolded), such that no matter what behaviour it displays it always receives a negative reinforcement, it will stop responding entirely (Seligman, 1972; Abramson et al., 1978). In other words, the experience of being unable to control outcomes can be shaped by reinforcement schedules that are not dependent on the actions of the animal. Recall that non-contingent positive reinforcement shapes superstition (Exline and Wilt, 2023; Skinner, 1948), which we claim is central to the inception of the experience of free will. Which is to say, uncoupling premotor sequences and downstream actions from feedback can either shape a sense of control or lack thereof. On the other hand, it is possible to instill a sense of control over one’s environment or ability to make choices free from external forces when no such control exists by using techniques developed by professional magicians and other illusionists (Pailhès et al., 2021). Forcing techniques make use of priming, restriction, availability heuristics, emotional salience, and other psychological factors to cause predetermined outcomes while preserving the experience of free will (Pailhès et al., 2020). Together, these data support the claim that the experience of free will can be shaped by relatively simple forms of manipulation including operant conditioning.

## Implications

The assumption of free will is rooted in our languages, ethics, politics, and legal systems. Without it, words like intention, shame, regret, and guilt take on entirely different meanings. For example, because the experience of “intending” to act occurs downstream of the neural patterns that predict outcomes, the word cannot accurately be used to describe planning or any other forward-looking executive function. Despite a pervasive and persistent experience of free will, under the model we have described, there is no causal power to “intention” in the conventional sense. Intention is, under the current model, an experience that is more closely related to anticipation or prediction of future events that will soon be realized. Similarly, under this model, the experience of regret is based upon the false belief that different behavioural outcomes could have been achieved given the same preconditions. In the same way that regret fails to capture the reality of causal determinism, the experience of pride associated with achieving a goal must be reevaluated with the understanding that other outcomes were not possible given the preconditions. The words we use to describe our own thoughts and actions are guided by the *experience* of free will rather than its veridical status. Still, it is quite likely that a robust experience of free will, while rooted in superstitious conditioning, promotes positive fitness outcomes for group animals (Foster and Kokko, 2009). Consistent with the interface theory of perception (ITP), the objects and structures of experience itself arise as solutions to the problem of representing a world that can be navigated to maximize fitness outcomes (Prentner and Hoffman, 2024). Just as proponents of ITP claim that the probability of perceived objects existing homomorphically “out there” in an external environment is almost certainly zero despite conferring real fitness payoffs, we claim that the experience of free will does not need to mirror cause-effect events in the environment to confer similar benefits.

In the legal context, distinctions are drawn between voluntary and involuntary actions as well as planning and spontaneity (Ladd, 1952). Implied is the notion that those who acted illegally could have behaved otherwise given the same set of preconditions—an intuition built on the experience of free will but an incoherent assumption under causal determinism. Under a deterministic framework, we argue that the inability to behave lawfully is always effectively a medical problem for which there may or may not be an immediate medical solution. Indeed, the evidence suggests that known neurological and neuropsychiatric disorders are deeply entwined with criminality (Modestin and Ammann, 1996; Tiihonen et al., 1993; Hodgins et al., 2008). Individuals who have sustained brain injuries, affecting their frontal lobes in particular (i.e., broad regions that encompass the premotor and motor systems), display a marked lack of inhibition that prevents rule-following behaviour in social contexts (Reverberi et al., 2005). Indeed, violent and criminal populations display a disproportionately high indication of frontal lobe injury (Hodgins et al., 2008; Reverberi et al., 2005; Brower and Price, 2001; Turkstra et al., 2003; Fazel et al., 2011)—in some studies, over half of the criminal population tested displayed physical signs of frontal lobe damage (Brower and Price, 2001; Turkstra et al., 2003). Similarly, individuals with fetal alcohol spectrum disorder (FASD), characterized in part by structural abnormalities of the frontal lobes (Nuñez et al., 2011; Spadoni et al., 2007), are overrepresented in the criminal justice system (Flannigan et al., 2018; Felson et al., 2008). Of course, frontal lobe disinhibition can also be achieved pharmacologically with GABA agonists such as benzodiazepines or ethanol (alcohol), which act preferentially upon frontal circuits, thus promoting disinhibition and unlawful behaviour (Felson et al., 2008; French and Maclean, 2006). It should be noted, however, that conduct disorders, personality disorders, or even subclinical issues with authority that are associated with criminal behaviour are no less a consequence of brain states determined by factors far beyond the individual.

The ethics of viewing rule-breaking behaviour as a medical problem can help guide solutions. Under the assumption of causal determinism, retributive justice needlessly increases suffering for offenders who could not have done otherwise, even if they believe they could have. If victims of circumstance and their internal decision-making apparatuses are treated as uncaused causes, they become multiply victimized—first as conscious witnesses to determined outcomes that increase suffering, then as perceived moral failures in the minds of others who assume they could have done otherwise, and finally as subjects experiencing guilt, shame, and regret. Behavioural modifications, which can include punishment, may provide solutions. However, systems must be designed to assess and monitor progress with outcomes contingent on rehabilitation. Of course, if a medical solution is not available, it may be desirable to quarantine violent criminals from the rest of the population in the interest of the group. In such cases, individuals quarantined from society ought to be treated as victims of circumstance, deserving of care rather than wanton punishment. If a realistic, cost-effective medical solution were available to promote rule-following behaviour, a rehabilitative approach would always be preferred over quarantine.

One way to view behavioural outcomes in the absence of free will is through the lens of activation energy. Gu et al. leveraged white matter tractography to construct a maximum entropy model of brain dynamics, demonstrating that brains are constrained by an energetic landscape such that the most probable functional states are defined by minimal energy (Gu et al., 2018). Given enough time, the full cognitive-behavioural range of an individual’s brain will be expressed; however, the required time for the most improbable events to occur might exceed average life expectancy. Instead, because context, state, reinforcement history, and neuroanatomy will bias certain responses over others, a limited range of behaviour is most likely to be displayed. That range of probabilistic outcomes, associated with frequently co-activated brain regions, may define the individual’s personality, which can be expanded under stress or when greater activation energy is provided. It would be consistent to suggest that some responses are so energetically unfavourable that, for most individuals, no amount of time or outcome-promoting factors would allow for spontaneous expression. These energetic barriers might be overcome after a marked change of brain structure (e.g., brain injury), extreme experiences (e.g., near-death experiences), or by transiently changing neurochemistry to lower activation thresholds for certain cognitive states and behaviours (e.g., the use of psychoactive drugs, manic episodes, etc.), given the existing state of preconditions.

If the experience of free will is illusory, should we behave as if it is not? Operating as if experiences are false when others are operating as if they are true might lead to fundamental conflicts in human interaction. Can a politician privately hold the view that voters operate under a misattribution of causality while drawing their legitimacy and authority from elections, or “the will of the people”? Still policies, institutions, and technologies may be developed without the assumption of free will to maximize human flourishing and minimize suffering. Distinguishing between the experience of free will and its veridical status may also benefit the field of psychiatry. That the experience of free will is likely integral to known dopaminergic pathways that can be targeted with pharmacology or cognitive-behavioural modifications should provide some clinical solutions related to intractable guilt, inappropriate regret, or persistent feelings of hopelessness. Can the scientific community maintain its ethical standard of informed consent if the experience of free will is illusory? If participation is motivated by determinants such as the individual’s reinforcement history, and is consistent with previously displayed behaviours and professed values, does the experience of consenting add value to the action of participating? And is it ethically relevant to consider whether experimenters’ and participants’ beliefs about free will are aligned? Perhaps the concept of autonomy should be preserved regardless of the experience of free will or its existence as such.

## Limits of the hypothesis

The major limitation of the hypothesis is that it is only relevant to human cognition and behaviour if free will does not exist. If humans are the conscious authors of their own decisions and actions, our postulate is false and the model we have presented to explain the experience of free will is simply unnecessary. The experience of free will cannot be a misattribution of causality if conscious intentions, desires, decisions, and actions hold real causal power. However, for reasons discussed elsewhere, explanations of free will should center on the phenomenal, not the noumenal.

## Conclusion

The question of why we feel free is relatively uninteresting without the assumption of causal determinism. If the conscious experience of free will is merely a reflection of reality, you feel free because you are free. However, if free will is a fundamentally incoherent idea, the pervasive and persistent existence of an experience of free will demands an explanation. The model we have delineated provides a general mechanism, rooted in functional neuroanatomy, that explains how the experience of free will emerges from a misattribution of causality and superstitious conditioning within a sensorimotor feedback loop. Ultimately, a belief in free will is formed that is projected outward to others as part of an extended theory of mind that guides social interaction, ethics, taboos, public policy, and law—the building blocks of communities and civilizations. Of course, the compatibilist view remains possible under the current model—particularly when one considers the possibility of extending ideas of self to include one’s environment. An organism-environment system, wherein no factor can be considered “external” to the system itself, could be viewed as generating decisions and actions without exogenous influence despite a preserved assumption of causal determinism. The present model suggests it may be necessary to develop an ecological framework for the study of free will and its experiential correlates. Because the proposed model is not strictly substrate-dependent and may hold true for any system that is conscious, makes predictions, and receives feedback from its own actions, it may be possible to condition equivalent illusions of free will in synthetic or artificially intelligent systems that meet these criteria.

## Data Availability

The original contributions presented in the study are included in the article/supplementary material, further inquiries can be directed to the corresponding authors.
